# Quantitative Trait Locus and Genetical Genomics Analysis Identifies Putatively Causal Genes for Fecundity and Brooding in the Chicken

**DOI:** 10.1534/g3.115.024299

**Published:** 2015-12-04

**Authors:** Martin Johnsson, Kenneth B. Jonsson, Leif Andersson, Per Jensen, Dominic Wright

**Affiliations:** * AVIAN Behavioural Genomics and Physiology Group, Dept of Biology, Linköping University, 58183 Linköping, Sweden; † Department of Surgical Sciences, Orthopaedics, Uppsala University Hospital, 751 85 Uppsala, Sweden; ‡ Department of Medical Biochemistry and Microbiology, Uppsala University, 751 23 Uppsala, Sweden

**Keywords:** chicken, QTL, eQTL, genetical genomics, fecundity, brooding, egg

## Abstract

Life history traits such as fecundity are important to evolution because they make up components of lifetime fitness. Due to their polygenic architectures, such traits are difficult to investigate with genetic mapping. Therefore, little is known about their molecular basis. One possible way toward finding the underlying genes is to map intermediary molecular phenotypes, such as gene expression traits. We set out to map candidate quantitative trait genes for egg fecundity in the chicken by combining quantitative trait locus mapping in an advanced intercross of wild by domestic chickens with expression quantitative trait locus mapping in the same birds. We measured individual egg fecundity in 232 intercross chickens in two consecutive trials, the second one aimed at measuring brooding. We found 12 loci for different aspects of egg fecundity. We then combined the genomic confidence intervals of these loci with expression quantitative trait loci from bone and hypothalamus in the same intercross. Overlaps between egg loci and expression loci, and trait–gene expression correlations identify 29 candidates from bone and five from hypothalamus. The candidate quantitative trait genes include *fibroblast growth factor 1*, and *mitochondrial ribosomal proteins L42* and *L32*. In summary, we found putative quantitative trait genes for egg traits in the chicken that may have been affected by regulatory variants under chicken domestication. These represent, to the best of our knowledge, some of the first candidate genes identified by genome-wide mapping for life history traits in an avian species.

Life history traits that affect individual fecundity and mortality during different life stages are intrinsically linked to evolution, since they make up components of fitness such as rate of reproduction and longevity. The heritability of life history traits within populations is expected, and found to be low (Mousseau and Roff 1987), since variation is removed by selection. Due to the low heritability, and the fact that fitness is influenced by many related traits, and is hence polygenic, the identification of genes affecting fitness components has proven to be problematic. However, knowledge of the molecular genes and genetic variants involved would open the way to population genetic analysis of selection signals, comparisons between populations, and mechanistic analysis of the pathways behind life history variation. Examples where alleles cause life history and fitness effects that have been identified by genetic mapping include horns in Soay sheep (Johnston *et al.* 2013), inversion polymorphism in *Mimulus guttatus* (Lowry and Willis 2010), and flowering time in *Arabidopsis thaliana* (Werner *et al.* 2005; El-Assal *et al.* 2001).

Fecundity, egg production and brooding behavior are vital components of fitness in the chicken. Domestication, particularly the late phase of breed improvement, has involved massive selection for production traits. Thus, the capacity to produce eggs affects a component of fitness in both wild and domestic conditions. Domestic layer chickens produce both more and larger eggs (Kerje *et al.* 2003), and, crucially, modern layer breeds lack brooding behavior, and will continually produce eggs without incubating them. This is ideal when production is a key factor and artificial incubation is available, but is obviously disastrous for offspring production under natural conditions. This selection, and hence exaggeration, of egg production in domestic chickens gives an excellent comparison point in contrast to the wild progenitor of the modern chicken—the Red Junglefowl. The wild by domestic paradigm makes traits selected under domestication tractable for genetic analysis, and the crossing of these different strains can maximize variation that we can then use to map and identify genetic regions [quantitative trait loci (QTL)], and genes affecting variability in these traits. We can use the large divergence between Red Junglefowl and domestics to map variants and study their function, interactions, and pleiotropic effects. While population genetics is required to know the role of these variants in natural populations, this approach allows one to isolate and study naturally occurring variants at the molecular level.

A major stumbling block in QTL analysis is that, despite the relative ease of identifying QTL, the identification of the actual genes and mutations is extremely challenging. First of all, the genomic resolution of linkage mapping is relatively poor, but can be improved by extended breeding designs. In this work, we use an advanced intercross (Darvasi and Soller 1995), where meiosis are accumulated over generations of interbreeding to improve mapping resolution. Relatively recently, a combination of QTL and expression QTL (eQTL) analysis has been used with some success (Klein *et al.* 2004; Le Bihan-Duval *et al.* 2011). A further refinement is to correlate actual gene expression with the phenotypic trait using the same animals in the same cross, enabling a rapid narrowing of potential candidate genes (Johnsson *et al.* 2012, 2014). The point here is to combine the evidence from trait–gene expression associations with genetic evidence from eQTL mapping to highlight candidate genes underlying the phenotypic QTL. The eQTL, or genetical genomics, approach allows the detection of quantitative trait genes for variants that affect the phenotype through changes in gene expression, assuming that measurements are taken from the relevant tissue and time point. It may also detect genes that are perturbed by variants, but are responsive, rather than causal, to the phenotypic effect, if they are also linked to the actual causal variant for the phenotype.

Egg production and bone allocation are intrinsically linked in the chicken, and in birds in general. When in lay, female birds form a special form of spongy bone in the medullary cavity. Medullary bone serves as a reservoir for calcium, and rapidly remodels during the laying cycle. The mobilized calcium is used for eggshell production, and is a major limiting factor in egg production. Also, structural cortical bone is lost during lay. Medullary bone content, and, to a more limited extent, cortical bone content, is therefore functionally connected with reproductive potential and egg production (Dacke *et al.* 1993). Furthermore, gene expression changes in medullary bone have been found to correlate with various egg production traits (Johnsson *et al.* 2012, 2014).

Here we report a large combined QTL and eQTL study using an advanced intercross line of a wild × domestic intercross chickens. We use two subsequent laying trials with and without egg removal to measure adult female egg fecundity and an as indicator of brooding behavior. By combining egg production QTL with local, putatively *cis*-acting, bone eQTL, we first identified initial candidates, and by then correlating the expression of each individual gene candidate with the corresponding overlapping fecundity phenotype, we generated 26 strong candidates for fecundity-related traits.

## Materials and Methods

### Cross design

The advanced intercross of Red Junglefowl and White Leghorn is based on one Red Junglefowl rooster and three White Leghorn females from the L13 line. The initial F_2_ intercross was expanded to an eighth generation advanced intercross. More detail about the intercross is given in Johnsson *et al.* (2012). Chickens were housed on three levels, with free access to food, perches and water; 572 individuals were genotyped at 652 single nucleotide polymorphism (SNP) markers using an Illumina Golden Gate assay by the Uppsala Seq & SNP platform. A full list of the markers is available in Johnsson *et al.* (2015). Chickens were reared in five separate batches.

### Phenotyping and QTL mapping

One month before slaughter at 212 days (around 26 weeks of age), a total of 232 hens were tested individually in two fecundity trials (see summary statistics in Supporting Information, Table S3, Table S4, and Table S5). In the first 2-wk trial, eggs were collected daily. In the second 10-d trial, aimed at measuring brooding, hens were given two dummy eggs, and were allowed to keep laid eggs. We calculated total and mean egg mass, and the number of eggs produced. The difference in number of eggs between the first and second trial is used as a measure of brooding, since a brooding hen will stop laying once a full clutch size is produced, when she is permitted to retain the eggs. Because the brooding trial was 4 d shorter than the fecundity trial (except in one batch), and to make the brooding score more interpretable, we extrapolated the number of eggs in the brooding trial up to 14 d before comparing it to the fecundity trial. Females that laid no eggs were excluded from analyses (11 from the fecundity trial, and 55 from the brooding trial). Chickens were tested in a total of five batches. In the case of the first two batches, as these exceeded the number of individual cages available, testing was staggered in two subbatches. This was then included as a covariate in subsequent analyses. Subsets of the fecundity data have previously been used to test for pleiotropic effects with relative comb mass (Johnsson *et al.* 2012, 2014). Brooding QTL are published in Henriksen *et al.* (unpublished data 2015). The current paper reports the full analysis including all hens and fecundity traits. We used R/qtl for QTL mapping (Broman *et al.* 2003). QTL mapping included body weight at slaughter (212 d) and batch as additive covariates. We used a principal component analysis (PCA) approach to control for any residual family structure (Wu *et al.* 2011). This was performed by calculating the first 10 PCs of all the genotype data, which were then tested for significance in each phenotypic QTL regression. Any significant PCs were then retained in the final model. This approach allowed us to both control for population substructure, and also test for epistatic interactions. We performed QTL mapping using both single-QTL scans and two-dimensional for epistatic pairs, and summarized the results for each trait with multiple-QTL models. Significance thresholds were calculated by permutation. A suggestive significance level was calculated using a genome-wide 20% *P*-value cut-off (principally due to being more conservative than the standard suggestive threshold (Lander and Kruglyak 1995), which gave a LOD of ∼3.6 per trait, while the genome-wide 5% significance threshold was a LOD of ∼4.4. Confidence intervals were calculated using a 1.8 LOD drop technique (Manichaikul *et al.* 2006). Epistatic interactions were also assessed using similar permutation thresholds (20% suggestive, 5% significant), as per the guidelines given in Broman and Sen (2009). The thresholds were approximately as follows: full model ∼11, full *vs.* one ∼9, interactive ∼7, additive ∼7, additive *vs.* one ∼4.

### Bone phenotypes and eQTL

Bone QTL and eQTL data were previously used to map and search for quantitative trait genes for bone traits; see Johnsson *et al.* (2015) for further details about bone phenotyping, QTL and eQTL mapping. After culling at 212 d of age, femoral bones were dissected out. The right femur was measured for bone density, area, content and thickness traits with peripheral computerized tomography, while the left was used for gene expression. Cortical and medullary bone was separated by setting thresholds of above 1000 mg/cm^3^ for cortical bone, and between 150 mg/cm^3^ and 1000 mg/cm^3^ for medullary bone, measuring a diaphyseal (50% of femoral length), and metaphyseal (6% of femoral length) cross-section. For the phenotypic QTL, the sexes were analyzed separately, and only the female QTL were used in this study. We isolated total RNA, made double-stranded cDNA from polyadenylated transcripts, labeled the cDNA and hybridized to NimbleGen 12 × 135k microarrays. Fluorescent intensities were preprocessed with the Robust Multiarray Average (RMA) algorithm (Irizarry *et al.* 2003), which includes background correction and quantile normalization of probe-level data followed by summarization to probesets. We performed eQTL mapping with Haley-Knott regression in a 100-cM region around the genomic location of each probeset, using R/qtl (Broman *et al.* 2003), and set significance thresholds by permutation. This analysis detects local, putatively *cis*-acting eQTL. The bone eQTL dataset consists of measurements from 125 females. Probe positions are provided in Table S1.

### Hypothalamus eQTL data

We also reanalyzed hypothalamus eQTL data from 129 individuals, also from the eighth generation of the same cross (Johnsson *et al.* 2015). The hypothalamus was dissected out after culling at 212 d of age, frozen and stored as above. Again, total RNA was isolated and double-stranded cDNA was synthesized in the same manner as for the bone samples. In this case, hybridization to NimbleGen 12 × 135k microarrays was performed at NimbleGen Services (Reykjavik, Iceland). eQTL mapping is detailed in Johnsson *et al.* (2015). RMA preprocessing and eQTL mapping were performed as described above. As the hypothalamus dataset includes both males and females, not all females were part of the fecundity trials. Therefore, this dataset contains only 43 females with both fecundity traits and gene expression data. Probe positions are provided in Table S2.

### Candidate quantitative trait genes

We searched for candidate quantitative trait genes affecting egg production by means of changes in gene expression detectable in bone. First, we extracted genes that have local *cis*-eQTL confidence interval overlapping the confidence interval for an egg QTL. Then, we tested for an association between the fecundity trait and the expression level of the probeset in question using a linear model that also included body mass at 212 d and a batch covariate. We used a significance level of 0.05, adjusted by Bonferroni correction for the number of uncorrelated eQTL overlapping that particular QTL.

### QTL overlaps

We tested for *cis*-eQTL within 100 cM around the genomic location of the probeset. Genomic confidence intervals were 1.8-LOD drop intervals, expanded to the closest marker. We used the R package GenomicRanges (Lawrence *et al.* 2013) to compare and overlap confidence intervals based on physical coordinates in the chicken genome (version 2.1/galGal3). Plots were created with ggplot2 (Wickham 2009). We tested the significance of QTL–sweep overlaps by randomly placing nonoverlapping intervals of the same number and size on an interval the size of the sequenced chicken genome and counting the overlaps. The *P*-value was estimated from the empirical cumulative distribution function based on 1000 iterations.

### Data availability

The gene expression data supporting the results of this article are available in ArrayExpress under accession numbers E-MTAB-3141 and E-MTAB-3154 (http://www.ebi.ac.uk/arrayexpress/). Fecundity phenotypes and genotypes underlying QTL mapping are available in Figshare at http://figshare.com/s/487302c8951611e5a80d06ec4b8d1f61.

## Results

### Medullary bone content predicts total egg mass

**Figure 1:**
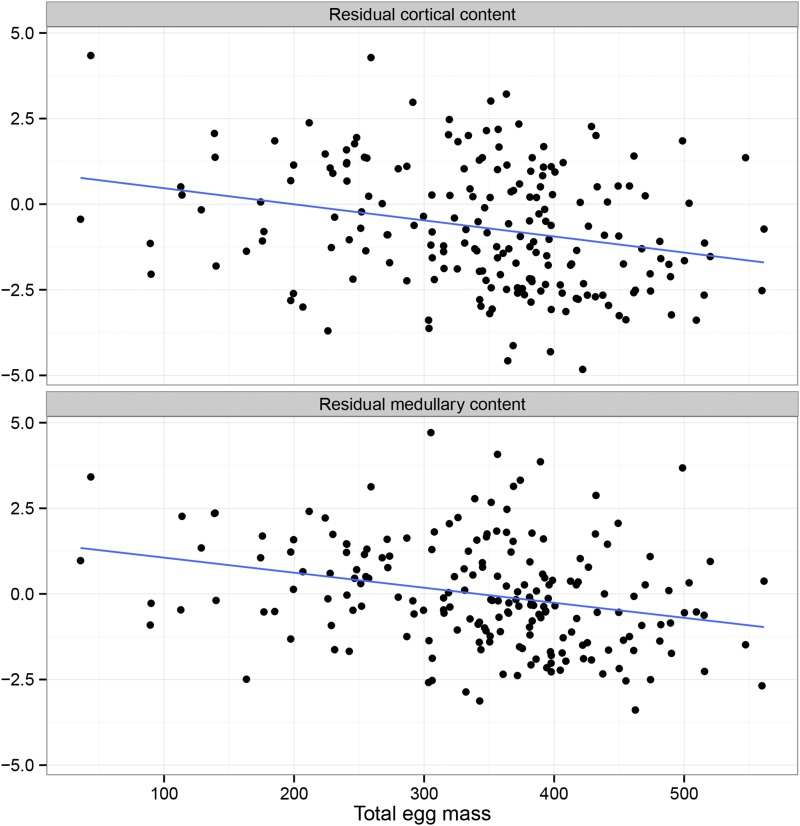
Scatterplot of the association between residual diaphyseal cortical and medullary content and total egg mass.

Total mass of eggs produced in the fecundity trial showed a negative correlation with medullary content (linear model with body mass at 212 d as covariate, *t* = –4.1, *P* = 5 × 10^−5^, *N* = 227) and cortical content (*t* = –3.7, *P* = 7 × 10^−4^), with this being inline with expectation given the relationship between fecundity and bone allocation, and also vindicating the choice of tissue for expression analysis (Figure 1). The difference between fecundity and brooding trial, our measure of broodiness, is not associated with these bone traits. This could be expected if this trait is driven by a behavioral change rather than direct correlation with bone characteristics.

### Fecundity QTL overlap QTL for bone and comb loci in the same cross

**Figure 2:**
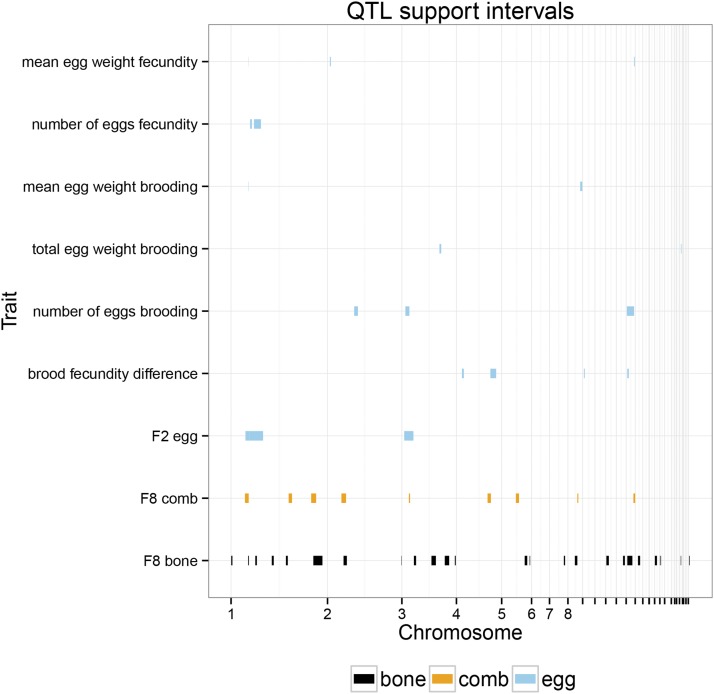
Genomic regions harboring QTL for egg traits, bone and comb. The *x*-axis displays physical distance on the autosomal chicken genome, with chromosomes in numeric order. The names of the microchromosomes have been suppressed.

We found a total of 12 QTL for different aspects of the fecundity trials, five for traits from the first fecundity trial, and seven for traits from the brooding trial (Table S5). There were also four QTL (already reported in R. Henriksen *et al.*, unpublished data for the difference between the fecundity and the brooding trial. When combined based on their genomic confidence intervals, they form 12 loci for egg traits (Figure 2). A comparison with previously published QTL for bone traits and relative comb mass showed that four fecundity QTL overlap loci for female bone traits, and four overlap with previously published relative comb mass loci.

**Table 1:** Egg QTL

Trait	Chr	Position (cM)	LOD	R2	Additive (SE)	Dominance (SE)	Covariates	Interactions
Mean egg weight brooding	1	515	11.74	10.6	2.32 +/− 0.45	1.50 +/− 0.51	w212, batch, part_fec, PC2,3,5,6	1@515.0:8@224.0
Mean egg weight fecundity	1	520	11.25	6.4	2.06 +/− 0.34	1.08 +/− 0.36	w212, batch, part_fec, PC2,3,5,8	
Number of eggs fecundity	1	591	6.74	11.7	−0.73 +/− 0.23	−0.56 +/− 0.32	w212, batch, part_fec, PC3,4	1@591.0:1@774.0
Number of eggs fecundity	1	774	3.78	6.3	−0.48 +/− 0.26	−0.33 +/− 0.35	w212, batch, part_fec, PC3,4	1@591.0:1@774.0
Mean egg weight fecundity	2	32.4	7.15	3.9	−0.97 +/− 0.31	−0.14 +/− 0.40	w212, batch, part_fec, PC2,3,5,8	2@32.4:13@212.9
Number of eggs brooding	2	467	10	17.8	0.95 +/− 0.42	1.86 +/− 0.64	w212, batch	2@467.0:13@193.0
Number of eggs brooding	3	111	3.96	6.5	1.81 +/− 0.44	0.06 +/− 0.77	w212, batch	
Total egg weight brooding	3	522	7.61	14.4	20.92 +/− 14.88	5.69 +/− 21.09	w212, batch, PC1,5,10	3@522.0:24@77.0
Brood fecundity difference	4	154	9	12.5	−3.74 +/− 0.83	3.25 +/− 1.42	w212, batch, part_fec, PC1,5,6	4@154.0:13@54.0
Brood fecundity difference	4	492	7.76	10.6	1.16 +/− 0.36	−0.65 +/− 0.48	w212, batch, part_fec, PC1,5,6	4@492.0:9@0.0
Mean egg weight brooding	8	224	6.68	6.7	1.58 +/− 0.40	0.29 +/− 0.62	w212, batch, part_fec, PC2,3,5,6	1@515.0:8@224.0
Brood fecundity difference	9	0	5.06	6.6	0.09 +/− 0.33	0.03 +/− 0.47	w212, batch, part_fec, PC1,5,6	4@492.0:9@0.0
Brood fecundity difference	13	54	9.98	14	−4.39 +/− 1.62	4.83 +/− 2.84	w212, batch, part_fec, PC1,5,6	4@154.0:13@54.0
Number of eggs brooding	13	193	5.9	9.9	0.96 +/− 0.55	−0.28 +/− 0.73	w212, batch	2@467.0:13@193.0
Mean egg weight fecundity	13	213	7.59	4.2	1.14 +/− 0.32	0.07 +/− 0.41	w212, batch, part_fec, PC2,3,5,8	2@32.4:13@212.9
Total egg weight brooding	24	77	6.8	12.7	−6.94 +/− 16.79	−36.02 +/− 21.99	w212, batch, PC1,5,10	3@522.0:24@77.0

Quantitative trait loci (QTL) for fecundity phenotypes in the F_8_ advanced intercross. Genomics locations refer to the F_8_ genetic map. Positive additive effects mean larger trait values for the White Leghorn genotype. The dominance coefficient is the deviation of the heterozygote from the additive expectation, which is the midpoint between homozygotes. Hence, a positive dominance coefficient means that the heterozygote is closer to the White Leghorn genotype homozygote, and vice versa. The interactions column indicates pairwise epistatic interactions between QTL.

### Candidate genes for fecundity QTL

**Figure 3:**
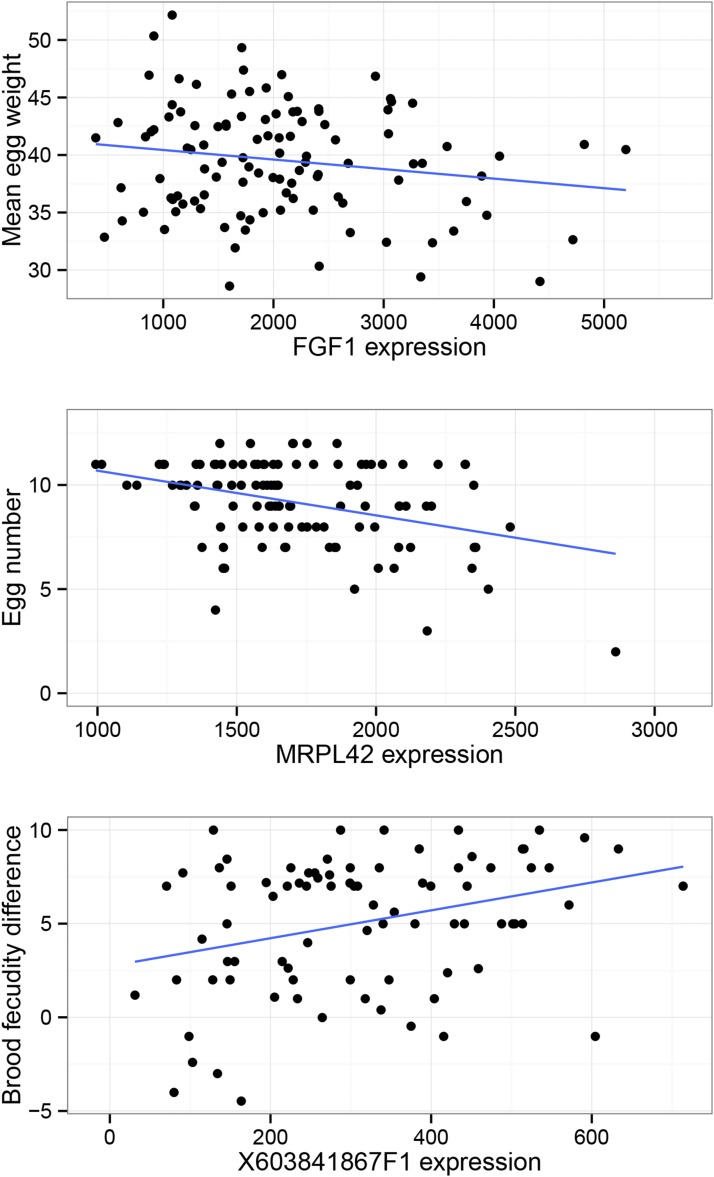
Scatterplots of gene expression and trait values of selected candidates: *FGF1*, *MRPL42*, and *603841867F1* expression *vs.* mean egg weight, egg number in the fecundity trial, and brood fecundity difference.

Given that fecundity QTL are mediated by genetic effects on bone, we were able to use the expression QTL we identified in bone tissue to assess the genes present within QTL confidence intervals. This was performed in a two-step process: first, we overlapped the egg QTL with eQTL from bone and kept those genes with overlapping eQTL as positional candidates. Second, we tested for an association between trait value and the expression level of each candidate gene. We found that a total of 1367 local eQTL overlapped with fecundity QTL (excluding the three QTL regions broader than 10 Mb reduces this number to 1035). Out of these, 26 eQTL genes both had a QTL–eQTL overlap and also significantly correlated with the overlapped fecundity trait. For many of the QTL, we found no such candidates in the bone eQTL data, and, for a few QTL, we found multiple associated genes (see Table 2 and Figure 3).

**Table 2:** Candidate genes from bone

Trait	Probeset	Chr	Location (Mb)	LOD	*P*-Value	*R*^2^
Number of eggs fecundity	ENSGALT00000018422_ENSGALG00000011293	1	46.8	2.8	0.00056	0.14
Mean egg weight fecundity	ENSGALT00000030673_ENSGALG00000019356	1	37.2	4.6	0.00144	0.48
Number of eggs brooding	X603222429F1	2	57.1	3.5	0.00070	0.16
Number of eggs brooding	X603953984F1	2	57.1	3.1	0.00035	0.18
Number of eggs brooding	X603157909F1	2	57.0	3.2	0.00104	0.16
Number of eggs brooding	X603469332F1	2	57.0	3.0	0.00011	0.20
Number of eggs brooding	X603371979F1	2	61.3	4.5	0.00071	0.16
Number of eggs brooding	X603234519F1	3	13.5	11.5	0.00012	0.20
Number of eggs brooding	ENSGALT00000014550_ENSGALG00000008947	3	14.8	9.9	0.00010	0.20
Number of eggs brooding	ENSGALT00000014548_ENSGALG00000008947	3	14.8	8.2	0.00046	0.17
Number of eggs brooding	NM_001199409_PAK7	3	14.8	10.6	0.00076	0.16
Number of eggs brooding	X603470949F1	3	9.9	7.1	0.00055	0.17
Number of eggs brooding	X603468747F1	3	15.6	4.7	0.00025	0.18
Number of eggs brooding	ENSGALT00000015469_ENSGALG00000009503	3	19.9	2.8	0.00018	0.19
Number of eggs brooding	X603841358F1	3	19.8	2.5	0.00111	0.15
Total egg weight brooding	X603220651F1	3	68.9	3.5	0.00017	0.15
Total egg weight brooding	X603848593F1	3	68.9	3.6	0.00005	0.17
Total egg weight brooding	ENSGALT00000025568_ENSGALG00000015858	3	80.7	2.6	0.00022	0.15
Total egg weight brooding	ENSGALT00000031669_ENSGALG00000015860	3	80.8	5.4	0.00096	0.12
Total egg weight brooding	X603371690F1	3	84.3	3.8	0.00087	0.13
Brood fecundity difference	X603841867F1	4	23.1	8.0	0.00191	0.10
Mean egg weight fecundity	NM_205180_FGF1	13	17.9	3.4	0.00083	0.48
Mean egg weight fecundity	ENSGALT00000011880_ENSGALG00000007343	13	17.9	3.0	0.00043	0.49
Total egg weight brooding	X603219553F1	24	2.6	7.8	0.00336	0.11
Total egg weight brooding	X603844217F1	24	2.6	3.2	0.00137	0.12
Total egg weight brooding	X603230423F1	24	5.2	4.8	0.00321	0.11

LOD scores refer to local *cis*-eQTL in the bone dataset. The *P*-values for trait-gene expression association and coefficients of determination refer to a linear model including body mass at 212 d and batch as covariates.

In particular, there are four QTL that have one such candidate gene each. These are QTL for egg number on chromosome 1 with *mitochondrial ribosomal protein L42* (*MRPL42*; *ENSGALG00000011293*), mean egg mass with an uncharacterized protein from the Ensembl gene database (*ENSGALG00000019356*) also on chromosome 1, a probeset based on an unknown EST (*603841867F1*) on chromosome 4, and *fibroblast growth factor 1* (*FGF1*; *NM_2*05180) on chromosome 13.

### Candidate genes from hypothalamic gene expression

The hypothalamus links the endocrine and nervous systems, and is therefore a plausible site of nervous system-dependent effects on fecundity traits. We therefore investigated a hypothalamus eQTL data set from the same advanced intercross in search of candidate quantitative trait genes (Johnsson *et al.* 2015). Out of a total of 79 hypothalamic eQTL overlapping fecundity QTL, five had a correlation between the trait value and gene expression value (Table 3). The most significant association was number of eggs in the brooding trial with *mitochondrial ribosomal protein L32* (*MRPL32*; *ENSGALG00000012337*) on chromosome 2.

**Table 3:** Candidate genes from hypothalamus

Trait	Probeset	Chr	Position (Mb)	LOD	*P*-value	*R*^2^
Number of eggs fecundity	X603599019F1	1	42.1	4.5	0.0050	0.17
Number of eggs brooding	ENSGALT00000020160_MRPL32	2	51.3	7.8	0.0003	0.35
Number of eggs brooding	X603864309F1	3	7.6	12.4	0.0114	0.19
Brood fecundity difference	X603568189F1	13	0.9	4.1	0.0163	0.14
Mean egg weight fecundity	X603868338F1	13	13.2	4.1	0.0278	0.54

LOD scores refer to local *cis*-eQTL in the hypothalamus dataset. The *P*-values for trait-gene expression association and coefficients of determination refer to a linear model including body mass at 212 d and batch as covariates.

### Overlaps with selective sweeps

A total of 12 selective sweeps, as identified by Rubin *et al.* (2010), were located within the fecundity QTL, meaning that seven out of 16 QTL overlapped at least one sweep. All of these sweeps were detected solely in layer birds, rather than being general to all domestics; however, this overlap appeared to be nonsignificant (*P* = 0.54 by simulation).

## Discussion

In this study, we identified a number of QTL affecting fecundity—a vital aspect of life history. A total of 12 QTL were detected, explaining on average 9% of the variation in each trait. A measurement of brooding, potentially relating to clutch size, also yielded four QTL. Combining these QTL with gene expression analysis, and eQTL previously identified in this cross, yielded a number of candidate genes for fecundity in this cross. These represent, to the best of our knowledge, some of the first candidate genes identified by genome-wide mapping for life history traits in an avian species. The use of gene expression evidence provides far greater weight than a linkage or association study alone, where the resolution is often insufficient to identify causal genes underlying the QTL. Our approach can identify candidate quantitative trait genes that act through changes in gene expression in bone or hypothalamus. It could also detect genes that work in other tissues, but where a similar eQTL effect is also present in bone. Bone, and the eggshell gland of the chicken display similarities on the transcriptome level (Jonchère *et al.* 2010). It therefore seems likely that our analysis may capture such epiphenomenal correlations as well.

Life history traits are among some of the hardest to analyze. They have been shown to have some of the lowest heritability values of any trait type (Mousseau and Roff 1987), making the identification of genes responsible for variation in such traits proportionally scarcer in the genome. It is striking that relatively few QTL were identified for these traits, though, given the low heritability, it is possible that many of the loci are in fact identified in this cross. Given the sample sizes of the QTL (232 females) and eQTL (125 for bone) studies, effect size overestimation is to be expected (Beavis 1998). A striking feature is the different architectures for the two types of fecundity traits, when eggs were removed daily or allowed to remain with the birds for the duration of the trial. This may be construed as a gene by environment interaction. It also reflects the connection between behavior and egg production. It can also indicate some of the reasons why the heritabilities for life history traits are so low, and the identification of causal genes is so complex, with such G × E interactions complicating the repeatability of such phenotypes. Despite this, two QTL for number of eggs in the fecundity trail, and one for number of eggs in the brooding trial, overlap QTL for egg mass located on chromosome 1 and chromosome 3 identified in the F_2_ generation of the same intercross (Wright *et al.* 2006, 2010). The chromosome 3 QTL also colocalizes with *comb1*, the major locus for relative comb mass in this cross, where linked and pleiotropic effects on egg production were previously detected.

A classic issue in chicken breeding as well as in life history evolution is trade-offs between genetically correlated traits. Genetically correlated fitness components, in the natural setting, or components of performance traits, can constrain the selection response. For instance, a negative genetic correlation between egg weight and egg number could be expected simply due to resource limitations. In our QTL results, we do not observe an overlap of egg weight and egg number QTL, which would indicate antagonistic (or positive) genetic effects. Instead, the architectures appear independent. It is possible that there are smaller effect antagonistic variants not detected in our QTL analysis. Also, the selection used to develop the founder White Leghorn line was aimed at counteracting antagonism by selection and crossing (Liljedahl *et al.* 1979). The separate architecture we observe may be partly due to successful breed improvement.

Brooding behavior can be considered a form of parental investment, and, in this context, is also related to clutch size in birds. Chickens in natural conditions will typically lay between five and eight eggs prior to ceasing laying and commencing incubation behavior (Meijer 1995). By allowing chickens to retain their eggs, and providing them with stimulus dummy eggs, we can map loci that curtail egg production in comparison to the previous trial. This is an indirect measure of brooding. Any other factor that causes hens to lay fewer eggs during the second trial that took place 2 wk later risk being confounded with brooding. However, at this time hens were well past sexual maturation, and genetic effects on development time seem unlikely to confound the measurement. Clutch size is extensively studied in the context of natural populations, having, as it does, a large effect on fitness. To date, a study using a 50k chip design using a wild population of collared flycatchers has identified that a single significant QTL for clutch size represents the sole genetic evidence for putative gene regions or genes affecting this trait (Husby *et al.* 2015). In a similar vein, a genome-wide association study of clutch size and egg mass in wild great tits found no significant associations, but chromosome partitioning results were consistent with a polygenic architecture (Santure *et al.* 2013). When it comes to domestic chickens, genome-wide association studies have implicated loci for egg weight (Wolc *et al.* 2012) and egg number, age at first egg, and egg shell traits (Liu *et al.* 2011).

We identify a number of candidate genes for fecundity and brooding behavior in the cross. *FGF1* is the sole candidate for a mean egg weight QTL on chromosome 13 from the brooding test. Fibroblast growth factors are heparin-binding proteins that are involved in signaling in various stages of embryonic development, regulation of cell division, angiogenesis, and other processes. They interact with heparin sulfate, which is abundant in bone, and involved in the regulation of developmental processes. We therefore hypothesize that *FGF1* may act through either a bone-dependent or ovary-dependent mechanism to promote increased egg production. In addition, we find two mitochondrial ribosomal proteins, *MRPL42* (egg number in fecundity trial on chromosome 1), and *MRPL32* (egg number in brooding trial on chromosome 2), as candidates acting by means of expression in bone and hypothalamus, respectively. Our eQTL analysis of bone traits suggests another mitochondrial ribosomal protein (*MRPS18A*) as a candidate for a QTL for diaphyseal cortical thickness. Mitochondrial ribosomal proteins contribute to translation of the proteins encoded in the mitochondrial genome. These associations with bone and fecundity traits in multiple tissues raise new questions as to the function of mitochondrial translation in the chicken. Finally, a bone-dependent locus should probably not be expected for the brood fecundity difference. However, we did find one candidate quantitative trait gene in the analysis (*603841867F1*, an unknown EST), which is worthy of further investigation. Further mapping and sequencing efforts are needed to find causative variants.

Experimental intercrosses of wild and domestic animals are powerful study systems to map variants and mechanisms behind domestication traits. Most importantly, they maximize the phenotypic variation present in the experimental cross, therefore maximizing the potential to identify functional variants. However, selection pressures as well as environmental conditions differ between domestic and wild conditions. Therefore, the genes most important in a trait in the wild and in the laboratory may be different. We can still use experimental intercrosses to uncover variants and mechanisms involved in a trait. However, it remains to be seen whether the genes identified as being of relevance for interpopulation variation also affect intrapopulation variation. To determine this, it would require population genetic studies in natural populations. By identifying such putative loci, it will now be possible to use candidate genes studies of both association and gene expression in selected tissues to help ascertain such effects.

## Supplementary Material

Supporting Information
