# Supplementary material for: Quantitative Trait Locus and Genetical Genomics Analysis Identifies Putatively Causal Genes for Fecundity and Brooding in the Chicken
Source: G3 (Bethesda). 2015 Dec 4;6(2):311–9. doi: 10.1534/g3.115.024299 (PMC4751551; doi:10.1534/g3.115.024299)
Supplement: Supporting Information [file supp_6_2_311__index.html]

Quantitative Trait Locus and Genetical Genomics Analysis Identifies Putatively Causal Genes for Fecundity and Brooding in the Chicken — Supporting Information 

# Quantitative Trait Locus and Genetical Genomics Analysis Identifies Putatively Causal Genes for Fecundity and Brooding in the Chicken

## Supporting Information for Johnsson *et al.*, 2016

**Files in this Data Supplement:**

- Table S1 - Probe positions for bone tissue arrays. (.xlsx, 1,476 KB)
- Table S2 - Probe positions for hypothalamus tissue arrays. (.xlsx, 1,114 KB)
- Table S3 - Summary statistics for fecundity phenotypes. (.xls, 33 KB)
- Table S4 - Pairwise Pearson correlations between fecundity phenotypes. (.xls, 34 KB)
- Table S5 - Genomic confidence intervals in basepairs (on reference assembly 2.1/galGal3) for fecundity QTL. (.xlsx, 50 KB)
